# Case report: A co-existing case of ulcerative colitis and dysferlinopathy in a male patient

**DOI:** 10.3389/fmed.2024.1481780

**Published:** 2025-01-22

**Authors:** Limin Li, Qiong Yan, Mingming Deng, Muhan Lü, Tiejun Zhou, Xiaolin Zhong

**Affiliations:** ^1^Department of Gastroenterology, The Affiliated Hospital of Southwest Medical University, Luzhou, China; ^2^Department of Pathology, The Affiliated Hospital of Southwest Medical University, Luzhou, China

**Keywords:** ulcerative colitis, dysferlinopathy, DYSF mutation, muscle weakness, case report

## Abstract

Patients with inflammatory bowel disease (IBD) are at risk of developing malnutrition, severe dystrophic muscle weakness, and muscle atrophy. We present a case of a 33-year-old male patient who exhibited concurrent muscle atrophy, muscle weakness, diarrhea, and mucopurulent bloody stools. Notably, the patient reported that his household or family members experienced similar symptoms of muscle weakness, without any apparent neurological or psychiatric manifestations. The patient’s intestinal symptoms were most consistent with ulcerative colitis, while the muscle weakness originated from dysferlinopathy following a comprehensive diagnostic work-up. The patient was initiated on vedolizumab therapy and L-carnitine therapy, and his symptoms improved after 1 month of follow-up. This case study underscores the necessity for clinicians to maintain a high level of suspicion for the presence of concomitant diseases when encountering IBD patients with muscle weakness. This approach is essential for achieving an early diagnosis and treatment.

## Introduction

Inflammatory bowel diseases (IBDs), including Crohn’s disease (CD) and ulcerative colitis (UC), are characterized by persistent immune-mediated inflammation of the gut and are usually diagnosed in adolescence and early adulthood (1, 2). UC is characterized by diffuse, continuous inflammation of the colon extending from the rectum proximally (3). The majority of individuals diagnosed with ulcerative colitis (UC) suffer from recurrent episodes of rectal bleeding, frequent diarrhea, and/or an increase in stool frequency. They may also experience bowel urgency, fecal incontinence, and crampy abdominal pain. Some patients report symptoms such as fatigue, fever, dehydration, sarcopenia, and weight loss. Approximately one-quarter of those with UC also exhibit extraintestinal manifestations (EIMs), which can include arthritis, inflamed bile ducts, or eye inflammation (4). IBD patients often suffer from malnutrition due to factors, such as high metabolism, insufficient nutrient intake, and malabsorption and intestinal loss of nutrients; these factors can all contribute to muscle and weight loss.

Dysferlinopathy encompasses a group of rare muscular dystrophies caused by recessive mutations in the *DYSF* gene. Mutations in *DYSF* are associated with a wide spectrum of phenotypes, which ranges from asymptomatic elevated serum creatine kinase (hyperCKemia) to selective and progressive involvement of the proximal and/or distal muscles of the limbs (5). The two major phenotypes are limb-girdle muscular dystrophy type 2B (LGMD2B), now called LGMDR2 according to the new nomenclature (6), presenting with proximal weakness in the lower limbs, and Miyoshi muscular dystrophy-1 (MMD-1). Muscle weakness usually occurs in the teenage years or early adulthood. The detection of dysferlin deficiency in muscle tissue or blood and along with the identification of mutations in the *DYSF* gene, are the primary methods for diagnosing dysferlinopathy (7).

## Case report

**Figure 1:**
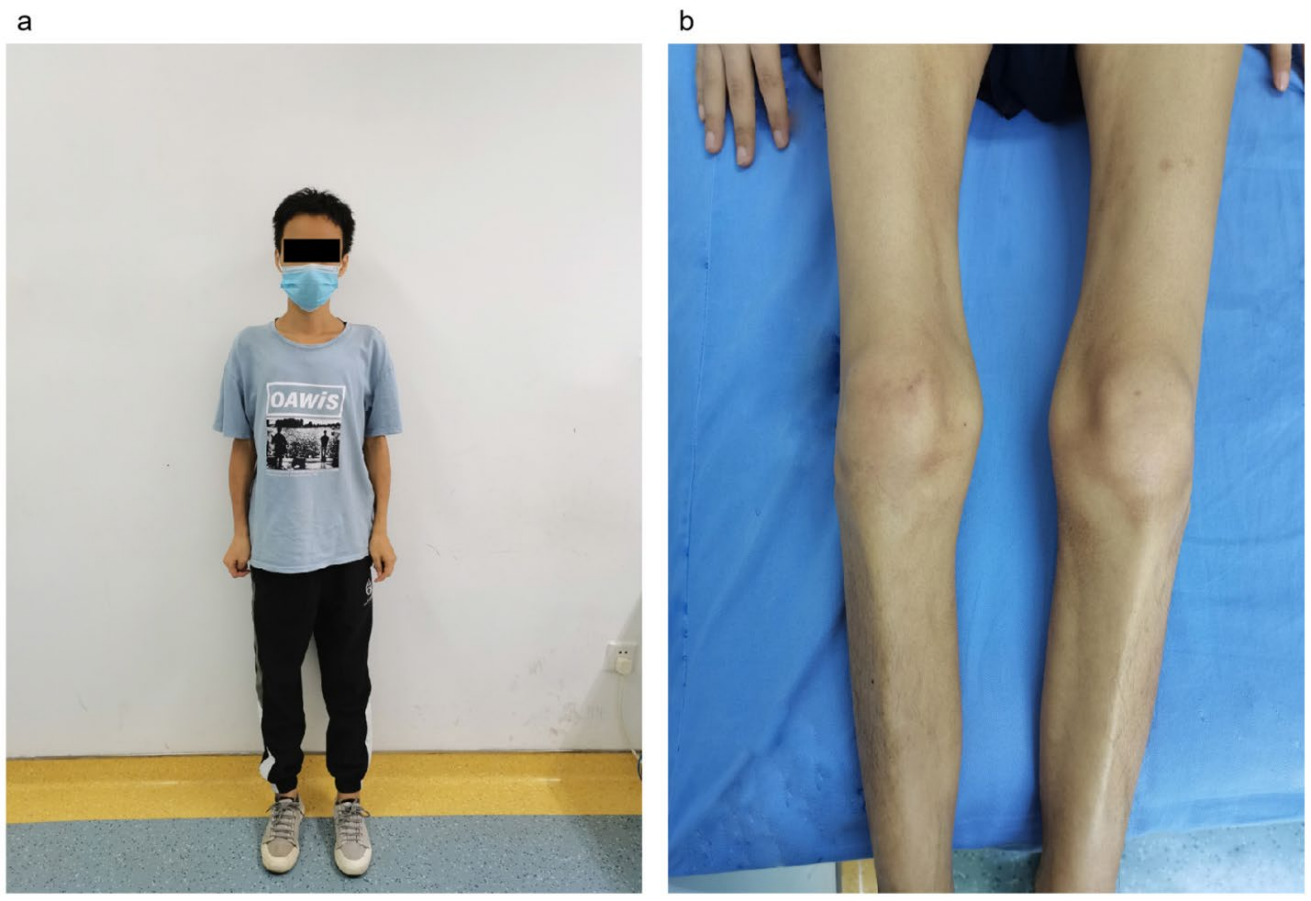
The condition of muscle weakness of the patient. **(A)** Overall condition of the patient, underweight. **(B)** Double lower limb muscular atrophy.

A 33-year-old male with no significant past medical history presented with a five-year history of weight loss and decreased muscle strength in the lower extremities (Figure 1), accompanied by 6 months of bowel symptoms, including mucopurulent blood stools and increased stool frequency. The patient was presented to our hospital, his height was 1.64 meters and weight was 41 kg, and his body mass index (BMI) score was 15.25 kg/m^2^. During this period, he had no history of abdominal pain or any change in mental status or behavior. The patient did not report taking any medications, and he had no history of tuberculosis treatment. He also stated that he neither smoked nor consumed alcohol.

**Figure 2:**
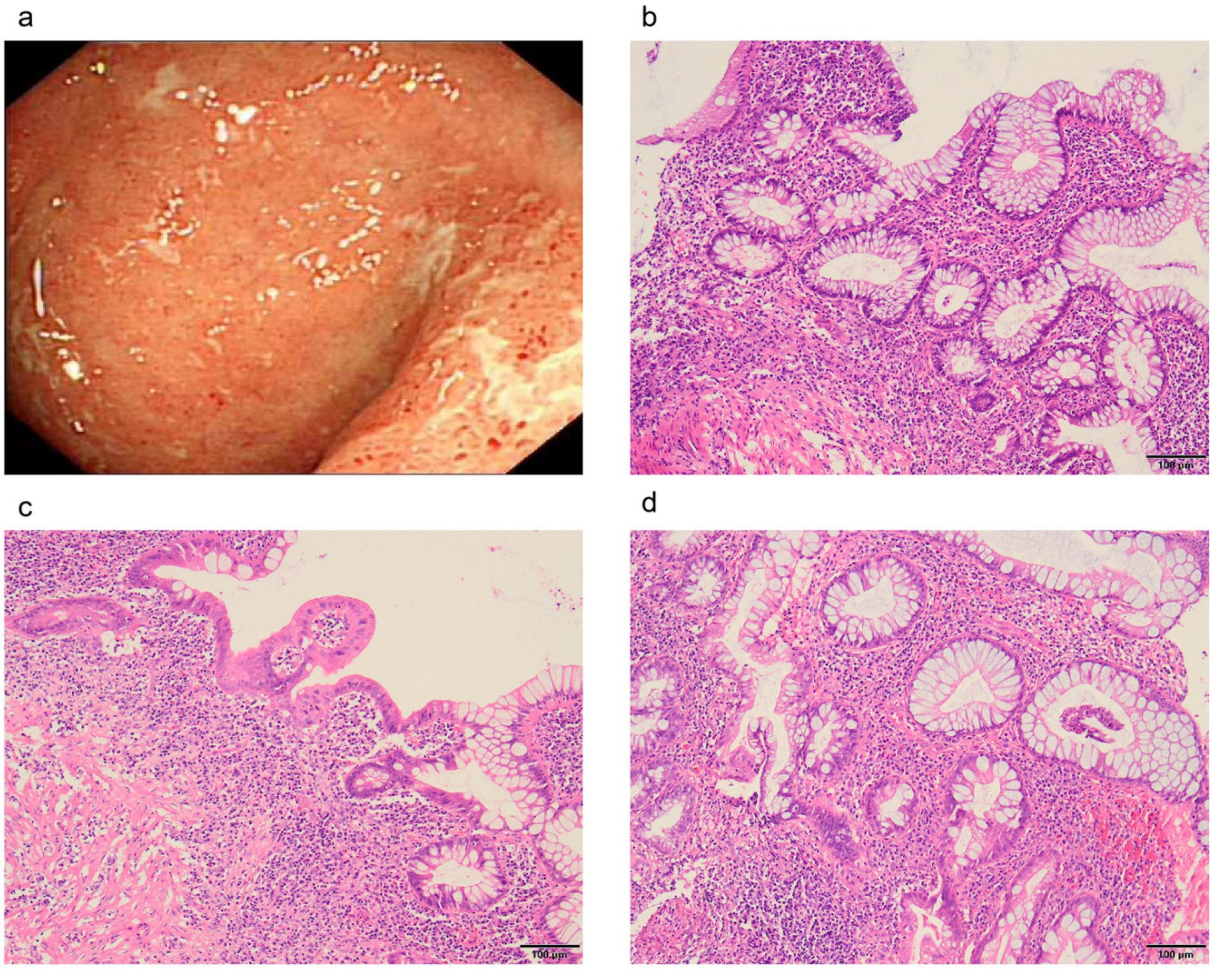
Colonoscopic and pathological manifestations of intestinal damage. **(A)** Inflammation and ulcers in the rectum. **(B)** Disturbed crypt structure with lymphocytic plasma cell sheet proliferation at the base of the mucosa. **(C)** The saphenous fossa is atrophied and reduced in number, with a widening of the spacing between the base of the saphenous fossa and the mucosal layer and a reduction in the epithelial cup cells in some of the saphenous fossae. **(D)** Crypt fossa branching, irregular.

On examination, the patient exhibited decreased muscle tone in all four limbs. The proximal muscle strength of both upper limbs was graded at 2, while the distal muscle strength of the right upper limb was graded at 4 and that of the left upper limb at 3–4. Proximal lower limb grade 2–3, distal lower limb grade 4, swaying lower limbs and hips when walking, limited forward flexion of the waist, generalized muscular atrophy of the skeletal muscles, and no pressure pain in all the muscles. The patient required external force to perform activities such as getting up and walking. The remainder of the physical examination yielded unremarkable results. We followed the guidelines for bowel preparation in patients with IBD and used polyethylene glycol to prepare the patient’s bowel, and electronic colonoscopy finally revealed colitis, and biopsies were taken from the ascending colon, transverse colon, descending colon, sigmoid colon and rectum. The basic laboratory workup is listed in Table 1. Based on the combined clinical evidence available to date, including but not limited to serology, endoscopy, and histopathology, the intestinal manifestations were ultimately caused by UC (Figure 2). To more accurately reflect the extent of the disease, we scored the patient’s endoscopic presentation with an Ulcerative Colitis Endoscopic Index of Severity (UCEIS) score of 6 and a Mayo Endoscopy Score (MES) score of 2. In addition, the patient’s symptoms should be classified as moderately active according to the partial Mayo score. However, due to the late onset of intestinal symptoms and markedly elevated muscle enzymes, we initially ruled out muscle dystrophy secondary to UC. It may cause by immune disorders or genetically inherited disorders. After an extensive literature review, we considered that this patient may have been diagnosed with myasthenia gravis (MG). However, the computed tomography (CT) scans and clinical manifestations did not yield supportive evidence for the diagnosis. To gain further clarity, a detailed history was obtained, during that the patient mentioned that other members of his household or family exhibited similar symptoms of muscle weakness. This led us to hypothesize that muscle atrophy could be a genetically inherited condition, prompting us to conduct genetic testing. The discovery of heterozygous mutations in the *DYSF* gene provided robust evidence supporting the diagnosis of dysferlinopathy. Treatment was initiated with intravenous administration of vedolizumab 300 mg and oral L-carnitine 1 g twice daily. The patient was also advised to enhance his nutritional intake. The treatment was aimed at improving the condition of IBD and promoted muscle cell metabolism. Following 1 month of treatment, a follow-up visit revealed a significant improvement in stool frequency, a weight gain of 1.5 kg, and a slight improvement in muscle weakness. The patient reported a significant overall improvement in symptoms and quality of life and was instructed to continue treatment with vedolizumab. The patient considered the treatment he received to be effective and was willing to follow up for a long period of time. The patient consented to publication of the case details.

**Table 1:** Laboratory workup performed on the patient at the time of presentation.

Laboratory measurement	Result	Normal range	Unit of measurement
White blood cell count	10.56^b^	3.5–9.5	×10^9^/L
Hb	54	130–175	g/L
PLT	782^b^	125–350	×10^9^/L
Creatinine	24.2	57–97	μmol/L
Alb	49.3	40–55	g/L
AST^a^	83.6^b^	15–40	U/L
ALT^a^	64.2^b^	9–50	U/L
LDH^a^	417.6^b^	120–250	U/L
ALP^a^	197.7^b^	45–125	U/L
CK^a^	2829.0^b^	50–310	U/L
ESR	28^b^	0–26	mm/h
PER	<3.00	25–280	ng/mL
IgG	17.5^b^	7.0–16	g/L
C3	1.07	0.7–1.4	g/L
C4	0.108	0.1–0.4	g/L
RF	Non-reactive		
ANA	Non-reactive		
cANCA	Weakly positive		
HBsAg	Non-reactive		
HCV Ab	Non-reactive		
EBV DNA	Non-reactive		
CMV DNA	Non-reactive		
TB-IGRA	Non-reactive		

RF, rheumatoid factor; ESR, erythrocyte sedimentation rate; Hb, hemoglobin; Alb, albumin; PLT, platelet.

a From muscles enzyme.

b Values out of normal range.

## Discussion

It is crucial to examine the potential reasons behind muscle weakness and atrophy in individuals diagnosed with IBD. To begin with, muscle weakness is linked to IBD itself, IBD patients suffer from intestinal absorption dysfunction and intestinal flora imbalance due to long term diarrhea, resulting in decreased nutrition absorption and bioavailability, which in turn disrupts muscle homeostasis and leads to clinical manifestations such as muscle atrophy and weakness (8). It is notable that severe muscular dystrophy is more prevalent in CD than in UC (9). Of course, muscle weakness also may be one of the extraintestinal manifestations (EIMs) of the condition (10), around 25% of individuals diagnosed with inflammatory bowel disease (IBD) experience extraintestinal manifestations (EIMs) prior to the onset of gastrointestinal symptoms. IBD patients also suffer from suboptimal levels of physical activity and sarcopenia (11), which depends on various factors both related to IBD (such as fear of flares) and personal factors (support from the partner or the patient’s social network). IBD is accompanied by a systemic increase in circulatory proinflammatory cytokines (9), inflammatory cytokines have a direct catabolic effect on protein metabolism, which reduce muscle protein synthesis and favor muscle protein breakdown, leading to muscle loss and sarcopenia (8). Secondly, the muscle weakness is secondary to other diseases, such as genetic or autoimmune disorders. The patient’s muscle weakness is familial aggregated and there is no history of alcoholism, malabsorption or drug abuse. Based on the patient’s history of muscle weakness and the results of genetic testing, dysferlinopathy was suspected and subsequently confirmed. Despite the administration of pro-muscle metabolism-related medications, there was no significant improvement in the patient’s condition.

The literature suggests that individuals combined with IBD and MG may have diminished muscle strength (12–15). MG is an autoimmune neuromuscular disease characterized by fluctuating fatigable muscle weakness (16). The distribution of limb weakness in MG is symmetrical and involves mainly the proximal muscle end (17). In this case, we ruled out MG based on the results of the chest CT scan and the clinical presentation.

The strength of this study is that it identified the first case of IBD combined with dysferlinopathy, and it also serves as a guide for future clinical practice: (1) When IBD patients combined with muscle weakness, it should be evaluated for the presence of it is also important to consider the possibility of a combination of other immune disorders or hereditary disorders, in addition to severe muscular dystrophies secondary to IBD. (2) When the diagnosis of the disease is unclear, it is essential to conduct further observation and analysis of the medical history.

## Data Availability

The data underlying this article are available in the article and in its online supplementary material.

## References

[1] Manichanh C Borruel N Casellas F Guarner F. The gut microbiota in IBD. Nat Rev Gastroenterol Hepatol. (2012) 9:599–608. doi: 10.1038/nrgastro.2012.152, PMID: 22907164

[2] Ni J Wu GD Albenberg L Tomov VT. Gut microbiota and IBD: causation or correlation? Nat Rev Gastroenterol Hepatol. (2017) 14:573–84. doi: 10.1038/nrgastro.2017.88, PMID: 28743984 PMC5880536

[3] Rosen MJ Dhawan A Saeed SA. Inflammatory bowel disease in children and adolescents. JAMA Pediatr. (2015) 169:1053–60. doi: 10.1001/jamapediatrics.2015.1982, PMID: 26414706 PMC4702263

[4] Voelker R. What is ulcerative colitis? JAMA. (2024) 331:716. doi: 10.1001/jama.2023.23814, PMID: 38306113

[5] Contreras-Cubas C Barajas-Olmos F Frayre-Martínez MI Siordia-Reyes G Guízar-Sánchez CC García-Ortiz H. Dysferlinopathy misdiagnosed with juvenile polymyositis in the pre-symptomatic stage of hyper CKemia: a case report and literature review. BMC Med Genet. (2022) 15:139. doi: 10.1186/s12920-022-01284-y, PMID: 35725460 PMC9208210

[6] Straub V Murphy A Udd B. 229th ENMC international workshop: limb girdle muscular dystrophies—nomenclature and reformed classification Naarden, the Netherlands, 17–19 March 2017. Neuromuscul Disord. (2018) 28:702–10. doi: 10.1016/j.nmd.2018.05.007, PMID: 30055862

[7] Fanin M Angelini C. Progress and challenges in diagnosis of dysferlinopathy. Muscle Nerve. (2016) 54:821–35. doi: 10.1002/mus.25367, PMID: 27501525

[8] Dhaliwal A Quinlan JI Overthrow K Greig C Lord JM Armstrong MJ. Sarcopenia in inflammatory bowel disease: a narrative overview. Nutrients. (2021) 13:656. doi: 10.3390/nu13020656, PMID: 33671473 PMC7922969

[9] Scaldaferri F Pizzoferrato M Lopetuso LR Musca T Ingravalle F Sicignano LL. Nutrition and IBD: malnutrition and/or sarcopenia? A practical guide. Gastroenterol Res Pract. (2017) 2017:8646495. doi: 10.1155/2017/8646495 28127306 PMC5239980

[10] Zaltman C Braulio VB Outeiral R Nunes T de Castro CL. Lower extremity mobility limitation and impaired muscle function in women with ulcerative colitis. J Crohns Colitis. (2014) 8:529–35. doi: 10.1016/j.crohns.2013.11.006, PMID: 24315794

[11] Gravina AG Pellegrino R Durante T Palladino G D’Onofrio R Mammone S. Inflammatory bowel diseases patients suffer from significant low levels and barriers to physical activity: the “BE-FIT-IBD” study. World J Gastroenterol. (2023) 29:5668–82. doi: 10.3748/wjg.v29.i41.5668, PMID: 38077160 PMC10701332

[12] Gondim Fde A de Oliveira GR Araújo DF Souza MH Braga LL Thomas FP. Two patients with co-morbid myasthenia gravis in a Brazilian cohort of inflammatory bowel disease. Neuromuscul Disord. (2014) 24:999–1002. doi: 10.1016/j.nmd.2014.06.434 25065584

[13] Guinet-Charpentier C Bilbault C Kennel A Perrier P Peyrin-Biroulet L Morali A. Unusual association of myasthenia gravis and ulcerative colitis in a 14-year-old boy. Arch Pediatr. (2015) 22:81–3. doi: 10.1016/j.arcped.2014.10.014, PMID: 25440769

[14] Rocha GV Furtado Leitão AM Gondim FAA. Myasthenia gravis, mediastinal goiter, and Crohn’s disease. Ann Thorac Surg. (2017) 103:2022. doi: 10.1016/j.athoracsur.2016.09.077, PMID: 28528037

[15] Finnie IA Shields R Sutton R Donnelly R Morris AI. Crohn’s disease and myasthenia gravis: a possible role for thymectomy. Gut. (1994) 35:278–9. doi: 10.1136/gut.35.2.278, PMID: 8307484 PMC1374509

[16] Punga AR Kusner L Berrih-Aknin S Le Panse R. Editorial: Advances in autoimmune myasthenia gravis. Front Immunol. (2020) 11:1688. doi: 10.3389/fimmu.2020.01688, PMID: 32983085 PMC7484602

[17] Meriggioli MN Sanders DB. Autoimmune myasthenia gravis: emerging clinical and biological heterogeneity. Lancet Neurol. (2009) 8:475–90. doi: 10.1016/S1474-4422(09)70063-8, PMID: 19375665 PMC2730933

